# Why do patients with radiation-induced sarcomas have a poor sarcoma-related survival?

**DOI:** 10.1038/bjc.2011.559

**Published:** 2011-12-15

**Authors:** B Bjerkehagen, M C Småstuen, K S Hall, S Skjeldal, S Smeland, S D Fosså

**Affiliations:** 1 Department of Pathology, Postboks 4953, NO-0424 Oslo, Norway; 2 Norwegian Radium Hospital, Postboks 4953, NO-0424 Oslo, Norway; 3 Oslo University Hospital, Postboks 4953, NO-0424 Oslo, Norway; 4 National Resource Centre for Late Effects, Postboks 4953, NO-0424 Oslo, Norway; 5 Department of Oncology, Postboks 4953, NO-0424 Oslo, Norway; 6 Department of Orthopaedic Surgery, Postboks 4953, NO-0424 Oslo, Norway; 7 Divisions of Surgery and Cancer Medicine, Postboks 4953, NO-0424 Oslo, Norway; 8 Institute for Clinical Medicine, University of Oslo, Postboks 4953, NO-0424 Oslo, Norway

**Keywords:** radiation, radiation-induced, sarcoma, secondary malignancies, prognosis, treatment-related morbidity

## Abstract

**Background:**

This study aims to provide reasons for the poor sarcoma-related survival in patients with radiation-induced sarcoma (RIS).

**Methods:**

We performed a case–control study comparing sarcoma-related survival of 98 patients with RIS to that of 239 sporadic high-grade malignant sarcomas.

**Results:**

The cumulative sarcoma-related 5-year survival was 32% (95% confidence interval (CI): 22–42) for patients with RIS *vs* 51% (95% CI: 44–58) for controls (*P*<0.001). Female gender, central tumour site and incomplete surgical remission were significantly more frequent among RIS patients than in controls. In multivariate analysis incomplete surgical remission (hazard ratio (HR) 4.48, 95% CI: 3.08–6.52), metastases at presentation (HR 2.93, 95% CI: 1.95–4.41), microscopic tumour necrosis (HR 1.88, 95% CI: 1.27–2.78) and central tumour site (HR 1.71, 95% CI: 1.18–2.47) remained significant adverse prognostic factors, but not sarcoma category (RIS *vs* sporadic).

**Conclusion:**

The poor prognosis of RIS patients are not due to the previous radiotherapy *per se*, but related to the unfavourable factors – central tumour site, incomplete surgical remission, microscopic tumour necrosis and the presence of metastases, the two former factors overrepresented in RIS.

Secondary sarcomas are defined as sarcomas developing after a previous cancer, with radiation-induced sarcomas (RISs) as a subgroup. The latter malignancies constitute ∼2.5–5.5% of all sarcomas (Huvos *et al*, 1985; Brady *et al*, 1992; Mark *et al*, 1994; Cha *et al*, 2004; Bjerkehagen *et al*, 2008; Gladdy *et al*, 2010). Most RISs are high-grade malignant by histology. The reported 5-year survival rates vary from 17 to 58% in RIS patients compared with 54–76% in patients with sporadic sarcomas (SPS) (Huvos *et al*, 1985; Lagrange *et al*, 2000; Singer *et al*, 2000; Cha *et al*, 2004; Thijssens *et al*, 2005; Bjerkehagen *et al*, 2008; Gladdy *et al*, 2010).

In a previous report, we identified incomplete surgical remission (defined as ‘no surgery’ or ‘contaminated margins’), microscopic tumour necrosis, metastases at diagnosis and central tumour site as prognostic factors for poor outcome in RIS patients (Bjerkehagen *et al*, 2008). These variables represent well-known prognostic factors also in SPSs (Singer *et al*, 2000).

Similar survival rates in RIS and SPS patients have been reported by several investigators in adjusted analysis of limited cohorts, provided that the patient was treated according to modern treatment (Bielack *et al*, 1999; Leclercq *et al*, 2004; Shaheen *et al*, 2006). However, a recent report on 130 patients with non-metastatic soft-tissue RISs concluded that previous radiotherapy was an independent poor prognostic factor (Gladdy *et al*, 2010). In our view, the published results of this large sample should be validated in other cohorts of RIS patients comprising both soft-tissue and bone sarcoma, as well as patients with metastases and those ineligible for radical surgery. Thus, the present study aims to investigate whether previous radiotherapy remains an independent prognosticator in unselected patients with RIS and in subgroups, if clinical and histological prognostic factors known to be valid in SPS are taken into account.

## Materials and methods

Our institutional sarcoma database contains consecutive patients referred to the Norwegian Radium Hospital from January 1980 and onwards (Aksnes *et al*, 2006). By April 2008, this database contained 3054 patients with soft-tissue sarcoma and 794 patients with bone sarcoma, covering >80% of the patients with sarcoma in the South–East region in Norway.

We searched the database for cases with a diagnosis of sarcoma and a previous history of radiotherapy. Our definition of RIS patients (cases) was based on slightly modified criteria presented by Cahan *et al* (1998), Arlen *et al* (1971) and Cha *et al* (2004) (Bjerkehagen *et al*, 2008):

1. The resulting sarcoma has developed within a prior radiation field.
2. A latency period of at least 2 years since the radiotherapy.
3. Histology compatible with high-grade malignant leiomyosarcoma (LMS), malignant fibrous histiocytoma (MFH), angiosarcoma (AS), malignant peripheral nerve sheath tumour (MPNST) and osteosarcoma (OS) different from that of the index tumour (Bjerkehagen *et al*, 2008). The selection of histological subtypes was based on the most frequent histological subtypes occurring among RIS.

Controls were patients with a SPS recorded in our database without previous radiotherapy. They were randomly selected among patients with sporadic high-grade malignant MFH, LMS, AS, MPNST and OS aiming for at least two controls per case, the histological subtype being the only criterion for matching. Uterine sarcomas were not included because no radiation-induced uterine sarcomas were identified.

**Figure 1:**
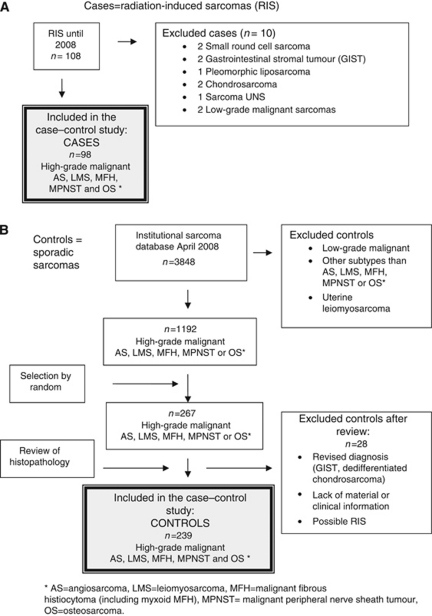
Flow chart showing the inclusion of patients in the case–control study including (**A**) radiation-induced sarcomas (cases, *n*=98) and (**B**) sporadic sarcomas (controls, *n*=239).

For the purpose of the study all histological specimens from the index tumours, the RISs and SPSs were reviewed by the first author. Several controls were finally excluded due to revised histological diagnosis, unknown primary tumour site, lack of histological material or insufficient clinical information (Figure 1).

For cases and controls, the following information was stored in a project database: gender, age at diagnosis of the sarcoma, histological subtype of sarcoma and index tumour, tumour size, presence of microscopic tumour necrosis, tumour site, tissue of origin (bone or soft tissue, the latter including viscera), metastases at presentation, local recurrence, complete surgical remission (defined as all tumour tissue removed with uncontaminated margins with at least 1 mm) at all sites, incomplete surgical remission (defined as operated with contaminated margins, primary tumour removed but metastases not excised or no surgery performed), the cause of not performing surgery and the date of sarcoma diagnosis (before 1990, 1990–1999, from 2000 and onwards). For cases, we also identified target radiation dose (Gy) and the latency from radiation to diagnosis of RIS.

The site of the sarcoma was categorised into two groups, based on the expected ease to perform radical surgery: (1) site in extremities (bone and soft tissue) + trunk wall (soft tissue including axillary region and groin) *vs* (2) central site: head and neck (bone and soft tissue), intrathoracic and intraabdominal (soft tissue and viscera), and axial skeleton including scapulae, clavicles, ribs and pelvic bones (Bielack *et al*, 2002).

Continuous variables were dichotomised as follows: age at diagnosis of the sarcoma <60 *vs* ⩾60 years (cut off based on median) and tumour size <5 cm *vs* ⩾5 cm.

Date and the cause of death were extracted from the Public Cause of Death Register in Norway.

### Ethical approval

The study was approved by the local ethical committee and permission was obtained from the Data Inspectorate of Norway.

### Statistical methods

Continuous variables were described with median and range. Some distributions were divided into categories based on clinical experience or structure of the data.

Associations between categorical variables were assessed with *χ*^2^-test. Sarcoma-related survival was evaluated by the Kaplan–Meier method, and survival distributions were compared using the log-rank tests. The observation time was calculated from the diagnosis of RIS or of SPS to the date of death or to the cut off date of the study (1 July 2009), whatever occurred first. Patients who died from causes other than sarcoma were censored at the date of death. The effect of prognostic factors on sarcoma-specific survival was modelled using Cox regression analyses. As the assumption of proportional hazards was violated for some of these factors weighted Cox regression was applied (Schemper, 1992; Schemper *et al*, 2009).

Prognostic factors were included in a multivariate weighted Cox regression analysis, if they achieved a *P*-value ⩽0.1 in the univariate analysis and/or if they were clinically considered to be associated with survival. Missing values led to exclusion of the patient in the analyses concerned. Two-sided *P*-values <0.05 were considered statistically significant.

Statistical analyses were performed using SPSS software (SPSS, Inc., Chicago, IL, USA) and R statistical software (http://www.r-project.org/).

## Results

### Cases

The database contained eligible 98 RIS patients (72 females, 26 males) and 267 controls (Figure 1, Table 1). Radiation-induced sarcoma had developed after a latency of median 14.3 years (range 2.2–60.5 years). The database did not contain any post-radiotherapy sarcoma diagnosed before a latency of 2 years. All cases had been treated with external beam radiation for their index tumour, and 11 patients had received additional brachytherapy (median 50 Gy, range 30–192 Gy). Twenty patients had received chemotherapy for the index tumour, containing alkylating agents, cisplatinum and/or doxorubicin in 16 cases.

The histological diagnoses of the index tumours were breast carcinoma (*n*=21), uterine carcinoma (*n*=13), testicular germ cell tumour (*n*=12), ovarian cancer (*n*=12), retinoblastoma (*n*=9), cervical cancer (*n*=7) and others (*n*=24) including two sarcomas (one small round-cell sarcoma consistent with skeletal Ewing's sarcoma and one sacral chordoma, both index tumours with distinctly different histology from the subsequent RIS).

### Cases *vs* controls

Significant differences emerged between RIS and SPS patients with regard to the site of the sarcoma, with 60% of the RISs localised centrally *vs* only 23% of the SPSs (*P*<0.001; Table 1). Fewer RIS patients underwent surgery compared with patients with SPS (RIS: 71% SPS: 90%, *P*<0.001). Of those operated, surgery was complete in fewer RIS than SPS patients (64% *vs* 77% *P*=0.034). Sixty-nine percent of SPS patients, but only 46% of the RIS patients achieved complete surgical remission (*P*<0.001). Radiotherapy was less often used in cases (22%) than in controls (33% *P*=0.046), with no inter-group difference emerging with regard to chemotherapy. More RIS than SPS patients experienced local recurrences after surgery (41% *vs* 17%, *P*<0.001). Except for the dominance of females, all other factors were similarly distributed between cases and controls. In particular, no difference was found with regard to the histological subtypes and for soft-tissue sarcoma *vs* bone sarcoma.

### Survival

**Figure 2:**
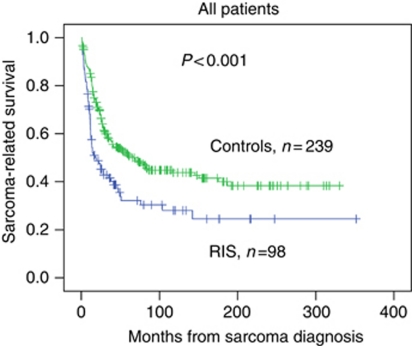
The sarcoma-related survival in patients with radiation-induced sarcoma compared with patients with sporadic sarcomas, Kaplan–Meier plot, log rank *P*<0.001.

*Radiation-induced sarcoma vs SPS* The median observation time was 103 months (range 10–352 months) for RIS patients and 191 months (range 19–330) for those with SPS. The sarcoma-related 5-year survival was 32% (95% CI: 22–42) for cases and 51% (95% CI: 44–58) for the controls, *P*<0.001 (Figure 2).

**Figure 3:**
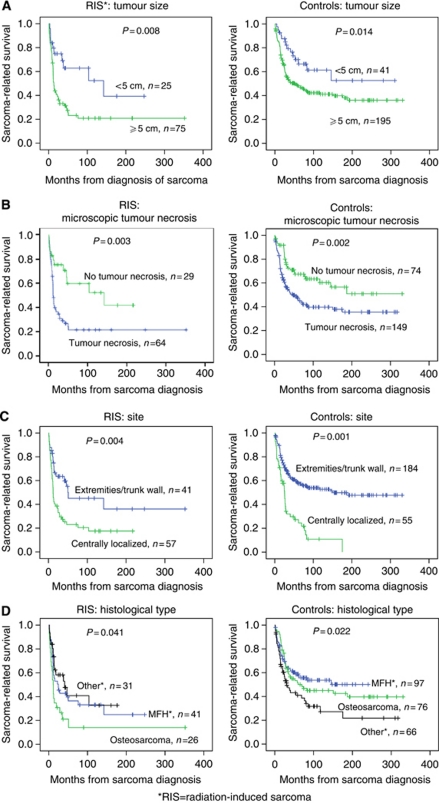
Kaplan–Meier plots showing sarcoma-related survival separating patients with radiation-induced sarcomas and sporadic sarcomas according to tumour size (**A**), presence of microscopic tumour necrosis (**B**), tumour site (**C**) and histology (**D**): malignant fibrous histiocytoma, osteosarcoma and others (malignant peripheral nerve sheath tumour, leiomyosarcoma and angiosarcoma).

*Radiation-induced sarcoma and SPS combined* Combining cases and controls in univariate analyses, factors significantly associated with favourable survival were: age <60 years at sarcoma diagnosis, tumour size <5 cm, no microscopic tumour necrosis, tumour site in the extremity and trunk wall, no metastases at diagnosis and complete surgical remission (Table 2). Survival differences with regard to tumour size, necrosis, site and histological type are depicted in Figure 3, separately for cases and controls.

In the multivariate analysis covering all patients those with incomplete surgical remission had a more than four times higher risk of dying from sarcoma (hazard ratio (HR) 4.48, 95% CI: 3.08–6.52). Metastases at diagnosis (HR 2.93, 95% CI: 1.95–4.41), microscopic tumour necrosis (HR 1.88, 95% CI: 1.27–2.78) and central tumour site (HR 1.71, 95% CI: 1.18–2.47) remained additional adverse prognostic factors for survival. Importantly, sarcoma category (RIS *vs* SPS) did not remain statistically significant.

In addition, multivariate analyses stratified for type of sarcoma (soft tissue excluding extraskeletal OS and OS in bone) were performed both for patients with and without metastases at presentation (Tables 3A and B). Sarcoma category (RIS *vs* SPS) did not remain a statistically significant prognostic factor for survival neither for OSs in bone nor for soft-tissue sarcomas. The presence of metastases and complete surgical remission emerged as significant prognostic factors for both the sarcoma types.

### Role of surgery

**Figure 4:**
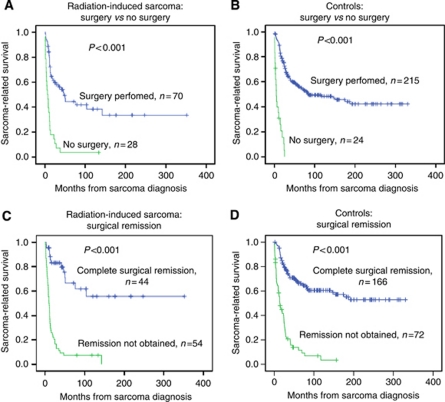
Kaplan–Meier plots showing sarcoma-related survival in patients in radiation-induced sarcomas and sporadic sarcomas according to performed surgery (**A** and **B**) and surgical remission (**C** and **D**) (incomplete surgical remission defined as no surgery at all or contaminated margins).

**Figure 5:**
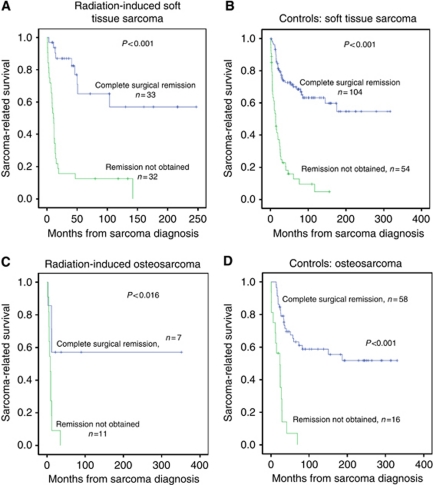
Kaplan–Meier plots showing sarcoma-related survival in patients with radiation-induced soft-tissue sarcomas (**A**), sporadic soft-tissue sarcomas (**B**), radiation-induced osteosarcoma in bone (**C**) and sporadic osteosarcoma in bone (**D**), and according to complete surgical remission.

The role of surgery was further investigated in subanalyses: the 5-year survival for cases and controls treated by any surgery was 44% (95% CI: 30–58) and 57% (95% CI: 50–64), respectively, (*P*=0.061). Independent of the group category, highly significant survival differences emerged between patients who received surgery compared with no surgery (Figures 4A and B). Complete surgical remission was of similar importance in patients with RIS and those with SPS, with a dismal prognosis in those without surgery at all or incomplete surgery (Figures 4C and D). In patients obtaining a complete surgical remission, 5-year survival rates were 67% (95% CI: 49–84) for RIS patients and 67% (95% CI: 59–75) for controls (*P*=0.874). For the patients with soft-tissue sarcoma who obtained complete surgical remission, the 5-year survival rates for radiation-induced soft-tissue sarcoma and sporadic soft-tissue sarcomas were similar, being 65% (95% CI: 44–86) and 71% (95% CI: 62–81) *P*=0.767, respectively (Figure 5).

More RIS patients were operated with positive margins than SPS patients, 36% *vs* 19%, respectively (*P*=0.003; Table 4). Furthermore, among patients with positive margins there were more local recurrences among RIS patients compared with controls, 72% *vs* 33% (*P*=0.002). For patients with positive margins, there was a non-significant difference in the use of postoperative radiotherapy, with fewer RIS patients treated with radiotherapy, 32% *vs* 53% (*P*=0.106). Regarding the effect of radiotherapy on local control in patients with positive margins, local recurrence occurred more often in RIS (4 out of 8) compared with SPS (7 out of 21; *P*=0.433).

Among patients who eventually died of sarcoma, there were more patients with local recurrence without registered metastases in RIS patients compared with SPS patients, 24% *vs* 11%, and there were fewer RIS patients with distant event (metastases) only compared with SPS patients, 29% *vs* 66%.

In 68% of operated RIS patients with tumour-positive margins (17 out of 25), the tumour was located centrally as compared with 35% (14 out of 40) in those with SPS (*P*=0.010).

There were 52 patients (29% of the cases and 10% of the controls) not treated with surgery. Among the RIS patients, the causes of omittance of surgery were local inoperability of the primary tumour (*n*=15), presence of metastatic disease (*n*=4), inoperable and metastatic (*n*=7), poor general medical condition (*n*=1) and unknown cause (*n*=1). For the controls, the causes were local inoperability (*n*=11), presence of metastatic disease (*n*=5), inoperability and metastases (*n*=3), patient refusal (*n*=2) and unknown cause (*n*=4).

## Discussion

In this case–control study of patients with sarcoma, significantly reduced survival was observed in patients with RIS compared with those with SPS. Sarcoma-related survival was, however, similar in patients with RIS or SPS, if they had achieved a complete surgical remission. The poor prognosis of RIS was not due to previous radiotherapy *per se*, but related to the unfavourable factors – central tumour site, incomplete surgical remission, microscopic tumour necrosis and the presence of metastases, the two former factors overrepresented in RIS patients.

For the subgroup of patients with soft-tissue sarcoma, metastases at presentation, incomplete surgical remission and microscopic tumour necrosis turned out to be significant prognostic factors. For patients with OS, metastases at diagnosis, incomplete surgical remission and central site were significant prognostic factors. Importantly, previous radiation did not remain an independent prognostic factor in any subgroups when adjusted for the above-mentioned variables.

Based on a case–control study on soft-tissue sarcoma, investigators from the Memorial Sloan-Kettering Cancer Centre (MSKCC) concluded that previous radiation represented an independent adverse prognostic factor among patients with soft-tissue sarcoma with a HR of 1.7 (Gladdy *et al*, 2010). This study and ours vary from each other with regard to some methodological aspects, which have to be considered:

First, Gladdy *et al* (2010) restricted their analysis to non-metastatic soft-tissue sarcoma, whereas we also included bone sarcomas and patients with metastatic RIS. However, even in a subanalysis of non-metastatic soft-tissue sarcomas, taking into account the four above mentioned independent prognostic factors, previous radiotherapy did not emerge as a prognostic factor. Second, in our series, all histological material in RIS and SPS patients was reviewed and our matching was based on histological types. This resulted in a similar distribution of the histological subgroups in our RIS and SPS patients. In the MSKCC study, it is not clear whether histological review was done for all control patients. Finally, our series includes a more unselected study population, also inoperable patients. The NRH is the only hospital treating sarcoma patients diagnosed in a defined area of Norway thus differing from the MSKCC, which is a tertiary referral hospital. We believe that much of the discrepancy between the results from the MSKCC series and our study can be explained by differences in patient selection and matching. Both the studies contribute to the understanding of the outcome in RIS patients, though the results should be interpreted on the background of the above inter-sample differences.

Other smaller studies support our findings of similar survival in RIS and SPS patients, when considering known prognostic factors. In a study of secondary OS including 17 RIS, Bielack *et al* (1999) concluded that, provided that local control is achieved, a patient with a secondary OS treated with modern combined modality therapy has a similar prognosis as patients with sporadic OS. These results are in accordance with subsequent reports from Leclercq *et al* (2004), Bacci *et al* (2007) and Shaheen *et al* (2006).

Though in our study we could not demonstrate that previous radiotherapy was an independent prognosticator in RIS, we cannot exclude that radiotherapy has led to the demonstrated clustering of unfavourable prognostic factors as evident for incomplete surgery and central tumour site. Radiotherapy may act as a mediator for biological changes, such as *MYC* amplification in some sarcoma subgroups (Manner *et al*, 2010; Guo *et al*, 2011).

An important observation in the present report is the higher number of patients with RIS not offered surgery compared with sporadic controls. In addition, a higher percentage of RIS patients were operated with positive margins, experienced local recurrences and fewer RIS patients than controls were offered radiotherapy. Furthermore, subanalysis of the patients who eventually died of sarcoma showed that significantly fewer RIS patients compared with SPS patients had received surgical treatment, but more RIS patients were operated with positive margins and had a higher percentage of local recurrences. All together the data indicate that local control is more difficult to achieve in RIS patients compared with SPS patients including the group of patients that received surgery. Most probably this is due to the more frequent central localisation of the tumour and the previous irradiation making radical surgery difficult or impossible.

Compared with SPS patients, a lower fraction of RIS patients received radiotherapy in combination with surgery. The limited use of radiotherapy in our sarcoma patients in general is explained by the previous restrictive attitude towards adjuvant radiotherapy according to the Scandinavian Sarcoma Group guidelines (Bauer *et al*, 2001). From 1986 to 1991, only 28% of sarcoma patients had radiotherapy. In 1998, the guidelines were changed increasing the percentage of patients receiving radiotherapy to 53% (Jebsen *et al*, 2008). In spite of this restrictive radiation policy, the 5-year survival for sporadic MFH is similar in the current series and MSKCC series (Gladdy *et al*, 2010). Admittedly re-treatment with radiotherapy remains a challenge in RIS, especially in centrally localised tumours. However, today's radiation techniques make adjuvant re-radiation possible and more frequent than in our retrospective series, thus, improving local control (McDonald *et al*, 2011).

In our study, cases were matched with controls for a single factor (histological type). Matching by gender, tumour site, age and year of diagnosis was not possible because of limited patient numbers in each histological subgroup. Instead, we performed adjustments for possible confounders in the multivariate analyses. Finally, we used *P*<0.05 as the limit of statistical significance in spite of multiple testing. However, we always present the exact *P*-values enabling the reader to make his/her own decisions regarding significance.

A major strength of this study is the completeness of the data regarding date and cause of death as the principal outcome based on the combination of registry data and information from the medical records. A second strength is the size – 98 cases of RIS patients are, to our knowledge, the second biggest material published (Gladdy *et al*, 2010). Another strength is that an experienced sarcoma pathologist reviewed all the histological slides of both the cases and controls.

In conclusion, this case–control study shows that the poor sarcoma-related survival in RIS patients compared with those with SPS is primarily related to a clustering of central tumour site and incomplete surgical remission. Our results do not support the suggestion that previous radiotherapy is an independent prognostic factor in RIS, when the above prognosticators are taken into account. However, the role of radiotherapy as a mediator cannot be excluded. The high number of RIS patients who were not offered surgery and/or radiotherapy remains a clinical challenge. Radiation-induced sarcoma patients should be treated principally according to the same principles as other sarcoma patients, applying multimodal treatment with some limitation given by the previous therapy.

### Conflict of interest

The authors declare no conflict of interest.

## Figures and Tables

**Table 1:** Baseline characteristics of radiation-induced sarcomas (cases) and sporadic sarcoma (controls)

**Variable**	**RIS (*n*=98)**	**Controls (*n*=239)**	**Total (*n*=337)**	***P*-value**
*Gender*				
Female	72 (73%)	117 (49%)	189 (56%)	<0.001
Male	26 (27%)	122 (51%)	148 (44%)	
				
*Age at sarcoma diagnosis (years)*				
<60	51 (52%)	124 (52%)	175 (52%)	0.979
⩾60	47 (48%)	115 (48%)	162 (48%)	
				
*Time period of diagnosis*				
Before 1990	19 (19%)	65 (27%)	84 (25%)	0.294
1990–1999	35 (36%)	82 (34%)	117 (35%)	
From 2000	44 (45%)	92 (39%)	136 (40%)	
				
*Histological diagnosis* a				
Angiosarcoma	12 (12%)	18 (7%)	30 (9%)	0.637
MFH	41 (42%)	97 (41%)	138 (41%)	
Osteosarcoma	26 (27%)	76 (32%)	102 (30%)	
MPNST	7 (7%)	20 (8%)	27 (8%)	
Leiomyosarcoma	12 (12%)	28 (12%)	40 (12%)	
				
*Tumour size*				
<5 cm	25 (26%)	41 (17%)	66 (20%)	0.065
⩾5 cm	70 (74%)	195 (83%)	265 (80%)	
Unknown	3	3	6	
				
*Tumour necrosis*				
Yes	64 (69%)	149 (62%)	213 (67%)	0.729
No	29 (31%)	74 (31%)	103 (33%)	
Unknown	5	16	21	
				
*Bone or soft tissue/viscera*				
Bone	29 (31%)	81 (33%)	110 (33%)	0.595
Soft tissue/viscera	65 (69%)	158 (67%)	223 (67%)	
Unknown	4	0	4	
				
*Site* b				
Extremity/trunk wall	39 (40%)	184 (77%)	223 (66%)	<0.001
Head/abdomen/axial/thoracic	59 (60%)	55 (23%)	114 (34%)	
				
*Metastases at diagnosis*				
Yes	20 (20%)	39 (16%)	59 (18%)	0.370
No	78 (80%)	200 (84%)	278 (82%)	
				
*Chemotherapy*				
Yes	37 (38%)	106 (45%)	143 (43%)	0.257
No	60 (62%)	130 (55%)	190 (57%)	
Unknown	1	3	4	
				
*Radiotherapy*				
Yes	22 (22%)	78 (33%)	100 (30%)	0.046
No	76 (78%)	155 (67%)	231 (70%)	
Unknown		6	6	
				
*Surgery*				
Yes	70 (71%)	215 (90%)	285 (85%)	<0.001
No	28 (29%)	24 (10%)	52 (15%)	
				
*Complete surgical remission (operated patients only, n=70)*				
Yes	45 (64%)	165 (77%)	210 (74%)	0.034
No	25 (36%)	49 (23%)	74 (26%)	
Unknown		1	1	
				
*Complete surgical remission (all patients)* c				
Yes	45 (46%)	165 (69%)	210 (62%)	<0.001
No	53 (54%)	73 (31%)	126 (38%)	
Unknown		1	1	
*Surgery and radiotherapy*				
Yes	15 (15%)	67 (29%)	82 (25%)	0.01
No	83 (85%)	166 (71%)	249 (75%)	
Unknown		6	6	
				
*Local recurrences*				
Yes	29 (41%)	37 (17%)	66 (23%)	<0.001
No	41 (59%)	178 (83%)	219 (77%)	
No surgery	28	24	52	

Abbreviations: MFH=malignant fibrous histiocytoma; MPNST=malignant peripheral nerve sheath tumour; RIS=radiation-induced sarcoma.

a Localisation of the RIS: all angiosarcomas and MPNST were localised in soft tissue. In all 68% of the MFH, 92% of the LMS and 27% of the OS were localised in soft tissue.

b (1) Extremities (bone and soft tissue) + trunk wall (soft tissue including the axillary region and groin). (2) Head and neck (bone and soft tissue), intrathoracic and intraabdominal (soft tissue and viscera), and axial skeleton including the scapula, clavicle, ribs and pelvic bones.

c Complete surgical remission also includes patients not operated.

**Table 2:** Cox regression analysis of sarcoma-related survival in patients with radiation-induced sarcoma (cases) and sporadic sarcomas (controls)

		**Univariate analysis**	**Multivariate weighted Cox regression^a^**		
**Prognostic factor**	***N*=337**	***P*-value**	***P*-value**	**HR**	**95% CI for HR**
*Radiation-induced sarcoma*		0.001		0.84	0.58–1.22
Yes	98 (29%)		0.362		
No	239 (71%) ref				
					
*Gender*		0.540			
Female	189 (56%)				
Male	148 (44%)				
					
*Age at sarcoma diagnosis (years)*		0.02		1.30	0.94–1.79
<60	175 (52%) ref		0.109		
⩾60	162 (48%)				
					
*Time period of diagnosis*					
Before 1990	84 (25%) ref				
1990–1999	117 (35%)	0.941			
From 2000	136 (40%)	0.224			
					
*Histological subtype*					
Malignant fibrous histiocytoma	138 (41%) ref				
Osteosarcoma	102 (30%)	0.333			
Others	97 (29%)	0.132			
					
*Tumour size*				1.57	0.97–2.55
<5 cm	66 (20%) ref	0.002			
⩾5 cm	265 (80%)		0.067		
Unknown	6				
					
*Microscopic tumour necrosis*				1.88	1.27–2.78
Yes	213 (67%)		**0.002**		
No	103 (33%) ref	<0.001			
Unknown	21				
					
*Bone or soft-tissue tumour*					
Bone	110 (33%) ref				
Soft tissue/viscera	223 (67%)	0.821			
Unknown	4				
					
*Site*				1.71	1.18–2.47
Extremity/trunk wall	232 (69%) ref	<0.001	**0.004**		
Head/abdomen/axial/thoracic	106 (31%)				
					
*Metastases at diagnosis*				2.93	1.95–4.41
Yes	59 (18%)	<0.001	**<0.001**		
No	278 (82%) ref				
					
*Complete surgical remission*				4.48	3.08–6.52
Yes	210 (62%) ref	<0.001	**<0.001**		
No	126 (38%)				
Unknown	1				

Abbreviations: CI=confidence interval; HR=hazard ratio; ref=reference; RIS=radiation-induced sarcoma.

Significant values are given in bold.

a In the multivariate analysis 307 patients are included.

**Table 3:** Cox regression analysis of sarcoma-related survival in patients with radiation-induced sarcoma (cases) and sporadic sarcomas (controls)

**(A) Patients with soft tissue sarcoma^a^**						
	**All, *n*=204^b^**			**Without metastasis at diagnosis, *n*=178^b^**		
**Prognostic factor**	***P*-value**	**HR**	**95% CI for HR**	***P*-value**	**HR**	**95% CI for HR**
*Radiation-induced sarcoma*	0.543	0.87	0.56–1.35	0.212	0.72	0.44–1.20
Yes (ref)						
No						
						
*Metastases at diagnosis*	<0.001	0.34	0.19–0.61	Not relevant		
Yes (ref)						
No						
						
*Complete surgical remission*	<0.001	4.96	3.14–7.84	<0.001	5.38	3.35–8.65
Yes (ref)						
No						
						
*Microscopic tumour necrosis*	<0.001	0.39	0.23–0.66	<0.001	0.35	0.21–0.60
Yes (ref)						
No						

**(B) Patients with osteosarcoma in bone**						
	**All, *n*=81^c^**			**Without metastasis at diagnosis, *n*=63^c^**		
**Prognostic factor**	***P*-value**	**HR**	**95% CI for HR**	***P*-value**	**HR**	**95% CI for HR**
*Radiation-induced sarcoma*	0.902	0.95	0.38–2.34	0.758	0.86	0.33–2.22
Yes						
No (ref)						
						
*Metastases at diagnosis*	0.008	0.36	0.17–0.76	Not relevant		
Yes (ref)						
No						
						
*Complete surgical remission*	<0.001	5.11	2.38–11.00	0.04	4.74	1.67–13.46
Yes (ref)						
No						
						
*Site*	0.026	2.80	1.13–6.96	0.038	3.31	1.07–10.24
Extremity/trunk wall (ref)						
Head/abdomen/axial/thoracic						

Abbreviations: CI=confidence interval; HR=hazard ratio; ref=reference.

a Including MFH, angiosarcoma, malignant peripheral nerve sheath tumour and leiomyosarcoma, but not osteosarcoma in soft tissue.

b Gender, age at diagnosis, histology, time period at diagnosis, tumour size and site were not significant prognostic factors.

c Gender, age at diagnosis, time period at diagnosis, tumour size and microscopic tumour necrosis were not significant prognostic factors.

**Table 4:** Local relapse and primary radiotherapy in patients offered surgery

	**RIS^a^**	**Controls^b^**	
**Characteristics**	***n*=98**	***n*=239**	***P*-value**
Surgery performed	70/98 (71%)	215/239 (90%)	<0.001
Positive margins in operated patients	25/70 (36%)	40/215 (19%)	0.003
Local relapse in patients with positive margins	18/25 (72%)	13/40 (33%)	0.002
			
*Radiotherapy*			
- In patients with positive margins	8/25 (32%)	21/40 (53%)	0.106
			
*Local relapse*			
- In patients with positive margins and treatment with radiotherapy	4/8 (50%)	7/21 (33%)	0.433
			
*Local relapse*			
- In patients with positive margins and no radiotherapy	14/17 (82%)	6/19 (32%)	0.003

a RIS=radiation-induced sarcoma.

b Controls=sporadic sarcoma.
